# Datasets for correlation dynamics of cocoa production in South Western Nigeria

**DOI:** 10.1016/j.dib.2018.03.076

**Published:** 2018-03-22

**Authors:** S.O. Edeki, M.E. Adeosun, G.O. Akinlabi, O.M. Ofuyatan

**Affiliations:** a Department of Mathematics, Covenant University, Ota, Nigeria; b Department of Mathematics & Statistics, Osun State College of Technology, Esa-Oke, Nigeria; c Department of Civil Engineering, Covenant University, Ota, Nigeria

**Keywords:** Cocoa product, Correlation dynamics, Nigeria-economy

## Abstract

In the Nigeria economy, cocoa production has been of great importance. This buttresses the fact that cocoa as a product is the leading agricultural export of Nigeria, leaving the country currently as the world fourth largest producer of cocoa, after Ivory Coast, Indonesia and Ghana and the third largest exporter, after Ivory Coast and Ghana. Hence, there is need for the agricultural sector expansion, effective predictive models and reliable price mechanism. This article examines tonnes of cocoa production dataset of the Nigeria agricultural sector for the period of twenty-four (24) years spanning between 1993 to 2016. The Correlation dynamics examined includes the autocorrelation features as affected by the production rate within the considered time interval. The degree of similarity between the dataset and the corresponding lagged version of itself over successive time interval is measured using a serial correlation test while the results mostly favour negative correlation showing that large current values correspond to small values at the specified lag. These dataset can effectively serve as good candidate for agricultural product modelling in terms of forecasting.

**Specifications Table**

**Table:** 

Subject area	*Agricultural Sciences*
More specific subject area	*Cocoa production*
Type of data	*Table, Excel file, graph.*
How data was acquired	*Direct contact via statistical bulletin*
Data format	*Analysed, CSV comma delimited.*
Experimental factors	Performance of correlation dynamics in relation to Nigeria agricultural sector with effect on cocoa production
Experimental features	*Serial correlation test and the degree of correlation*
Data source location	*Statistical bulletin*, Nigeria
Data accessibility	*Within this data article.*

**Value of the data**

• The present data will be of great usefulness for the determinants of cocoa production in Nigeria.
• The dataset will help to know the trend and pattern of cocoa production output over the time.
• The dataset can be used for agricultural product modelling and forecasting.
• The dataset will aid in budget planning since cocoa production contributes immensely to the country's economy.

## 1 Data

**Fig. 1:**
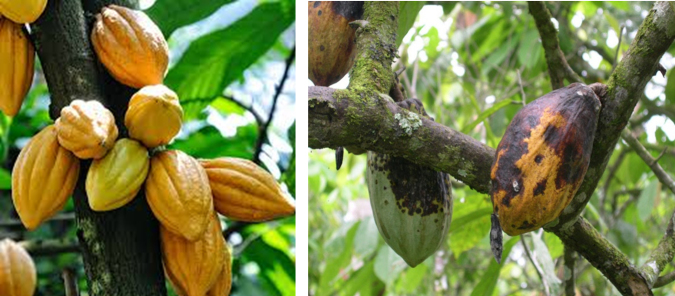
Health nature of cocoa product.

The datasets used in this work contains tonnes of cocoa production of the Nigeria agricultural sector for the period of twenty-four (24) years. This is presented in Table 1 followed by Fig. 1. Related literature on cocoa production and Nigerian economy includes the references in [1], [2], [3], [4], [5]. Predictive and forecasting approaches are of great importance in any production sector [6]. In addition to the mathematical formula (1), the data are processed/analysed via the statistical software (SPSS).

**Table 1:** Data on Cocoa Production in Tonnes (CPT).

S/N	Years	CPT	S/N	Years	CPT
1	1993	129.46	13	2005	194.13
2	1994	187.09	14	2006	122.14
3	1995	139.68	15	2007	111.09
4	1996	100.00	16	2008	172.35
5	1997	172.00	17	2009	168.00
6	1998	159.10	18	2010	167.79
7	1999	208.65	19	2011	188.07
8	2000	116.79	20	2012	123.08
9	2001	131.10	21	2013	120.33
10	2002	125.98	22	2014	132.50
11	2003	112.50	23	2015	144.80
12	2004	134.17	24	2016	112.80

Fig. 1 shows a graphical view of disease infested cocoa pods (Right), and non-infested cocoa pods (left). The healthy nature of cocoa trees have significant effect on the production of cocoa beans.

## 2 Experimental design, materials and methods

### 2.1 Design, and methodology

In addition to the statistical software used in the data analysis, is the mathematical model defined as follows:

(1) $$r_{1}=\frac{\sum_{\gamma =1}^{N-1}\left(x_{\gamma }-x_{1}^{m}\right)\left(x_{\gamma +1}-x_{2}^{m}\right)}{\sqrt{\sum_{\gamma =1}^{N-1}{\left(x_{\gamma }-x_{1}^{m}\right)}^{2}}\cdot \sqrt{\sum_{\gamma =1}^{N-1}{\left(x_{\gamma }-x_{2}^{m}\right)}^{2}}}$$

where $x_{1}^{m}$ and $x_{2}^{m}$ are the means of the first N−1 and the last N−1 observations respectively.

Eq. (1) represents the correlation coefficient computed between one time series and the same series lagged by one or more time units. Such correlation model is a good candidate for examining the relationship existing between adjusted volatilities in the market and the investment settings [7], [8], [9], [10].

### 2.2 Data analysis

**Fig. 2:**
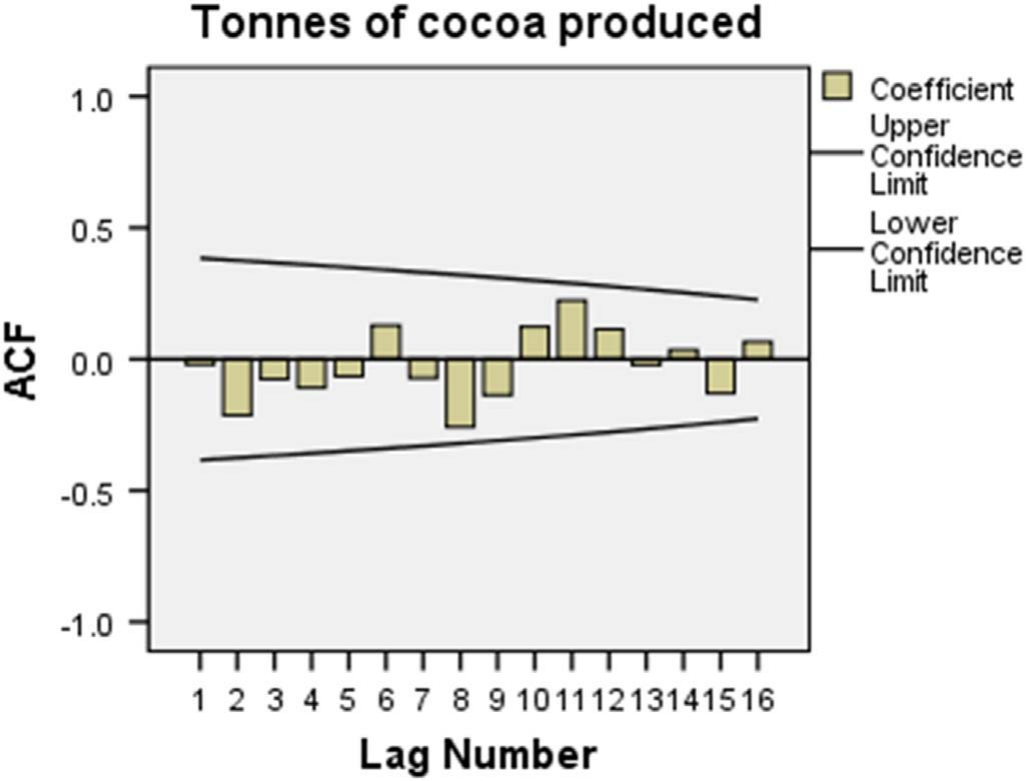
ACF plot for tonnes of cocoa produced.

**Fig. 3:**
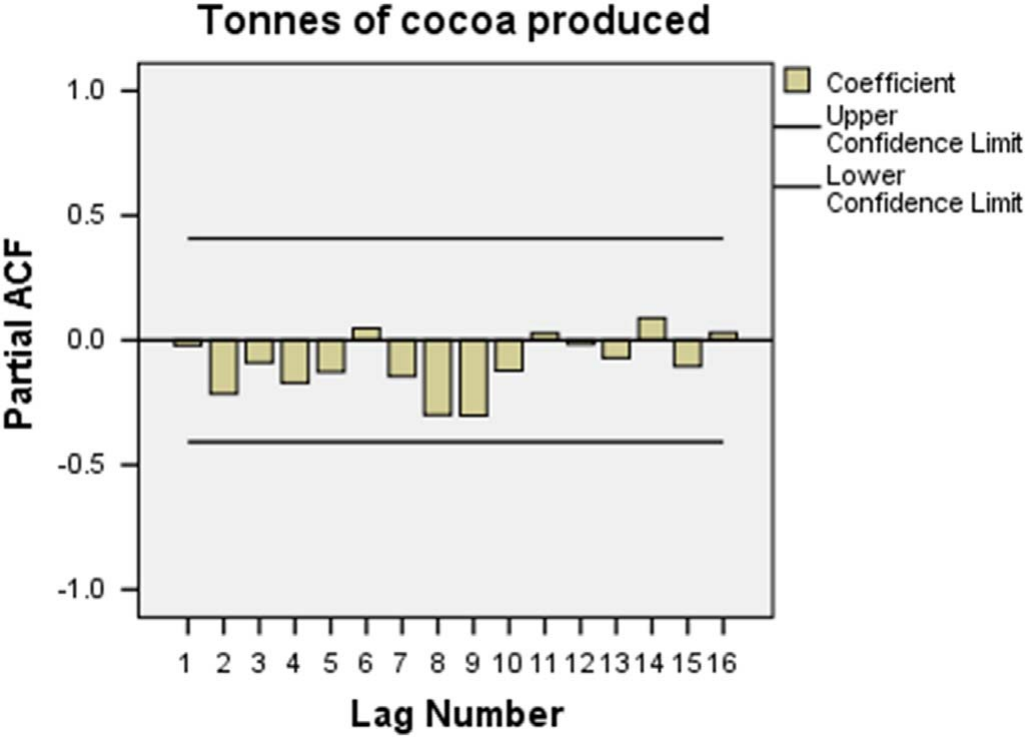
Partial ACF plot for tonnes of cocoa produced.

Here, the outcomes of the analysed data are presented in Table 2, Table 3, Table 4, Table 5, and Fig. 2, Fig. 3. ACF and PACF in this regard denote Autocorrelation Function and Partial Autocorrelation Function respectively.

**Table 2:** Description of the model.

Model Name		MOD_1
Series Name	1	Year
	2	Tonnes of cocoa produced
Transformation		None
Non-Seasonal Differencing		0
Seasonal Differencing		0
Length of Seasonal Period		No periodicity
Maximum Number of Lags		16
Process Assumed for Calculating the Standard Errors of the Autocorrelations		Independence (white noise)^a^
Display and Plot		All lags

Applying the model specifications from MOD_1.

a Not applicable (NA) for the standard errors calculation of the PACFs.

**Table 3:** Summary of case processing.

		Year	Tonnes of cocoa produced
Series Length		24	24
Number of Missing Values	User-Missing	0	0
	System-Missing	0	0
Number of Valid Values		24	24
Number of Computable First Lags		23	23

**Table 4:** Series: Tonnes of cocoa produced.

Lag	Autocorrelation	Std. Error^a^	Box-Ljung Statistic		
			Value	Df	Sig.^b^
1	−.021	.192	.012	1	.911
2	−.214	.188	1.311	2	.519
3	−.076	.183	1.482	3	.686
4	−.110	.179	1.858	4	.762
5	−.066	.174	2.001	5	.849
6	.129	.170	2.578	6	.860
7	−.072	.165	2.767	7	.906
8	−.258	.160	5.365	8	.718
9	−.136	.155	6.140	9	.726
10	.123	.150	6.810	10	.743
11	.224	.144	9.220	11	.602
12	.114	.139	9.892	12	.625
13	−.023	.133	9.921	13	.700
14	.035	.127	9.998	14	.762
15	−.130	.120	11.174	15	.740
16	.066	.113	11.514	16	.777

a The underlying process assumed is independence (white noise).

b Based on the asymptotic chi-square approximation.

**Table 5:** Partial Autocorrelations Tonnes of cocoa produced.

Lag	Partial autocorrelation	Std. error
1	−.021	.204
2	−.215	.204
3	−.090	.204
4	−.171	.204
5	−.127	.204
6	.048	.204
7	−.145	.204
8	−.300	.204
9	−.302	.204
10	−.122	.204
11	.029	.204
12	−.016	.204
13	−.071	.204
14	.089	.204
15	−.105	.204
16	.030	.204

### 2.3 Analysis overview

From the analysis, making reference to ACF in Fig. 2, it is pointed out that all the 16 coefficients are below the two-sided error limits. Only 6 out of the 16 are above the zero bar. From Fig. 3, the PACF shows that 12 out of the 16 coefficients are below the zero bar. Hence, there is a greater need to improve the trend model with regard to cocoa production in Nigeria. The ACF plot indicates significant autocorrelation and that the data are not stationary. Since stationary conveys the idea of the mean and standard deviation holding still and not shifting. The plot shows that the differenced data appear to be stationary and do not exhibit seasonality. Though, using regular differencing, the seeming trends can be adjusted by computing the difference between every two successive values.

## References

[1] Janek K. (2016). Dataset of cocoa aspartic protease cleavage sites. Data Brief.

[2] Egharevba M.E. (2016). Microfinance and poverty reduction strategy for promoting national development: the challenge of social/financial inclusion. Soc. Sci..

[3] Chidozie F.C., Peter O.I., Akande O.O. (2014). Foreign megastores and the Nigerian economy: a study of shoprite. Mediterr. J. Soc. Sci..

[4] Nwachukwu I.F. (2014). Dynamics of agricultural exports in sub-sahara Africa: an empirical study of rubber and cocoa from Nigeria. Int. J. Food Agric. Econ..

[5] Fawole W.O., Rahji M.A.Y. (2016). Determinants of productivity among farmers in Ondo State of Nigeria. AJAEES.

[6] Ofuyatan O.M., Edeki S.O. (2018). Dataset on predictive compressive strength model for self-compacting concrete. Data Brief.

[7] Edeki S.O. (2016). Parameter estimation of local volatility in currency option valuation. Int. Rev. Model. Simulations.

[8] Edeki S.O., Owoloko E.A., Ugbebor O.O. (2016). The modified Black-Scholes model via constant elasticity of variance for stock options valuation. AIP Conf. Proc..

[9] Pospíšil J., Sobotka T. (2016). Test datasets for calibration of stochastic and fractional stochastic volatility models. Data Brief..

[10] Edeki S.O., Ugbebor O.O., Owoloko E.A. (2016). On a dividend-paying stock options pricing model (SOPM) using constant elasticity of variance stochastic dynamics. Int. J. Pure Appl. Math..

